# Volatile Compounds and Biological Activity of the Essential Oil of *Aloysia citrodora* Paláu: Comparison of Hydrodistillation and Microwave-Assisted Hydrodistillation

**DOI:** 10.3390/molecules28114528

**Published:** 2023-06-02

**Authors:** Rafael M. Sprea, Luís H. M. Fernandes, Tânia C. S. P. Pires, Ricardo C. Calhelha, Pedro João Rodrigues, Joana S. Amaral

**Affiliations:** 1 Centro de Investigação de Montanha (CIMO), Instituto Politécnico de Bragança, Campus de Santa Apolónia, 5300-253 Bragança, Portugal; rafael.sprea@ipb.pt (R.M.S.); luiscellohenrique@gmail.com (L.H.M.F.); tania.pires@ipb.pt (T.C.S.P.P.); calhelha@ipb.pt (R.C.C.); 2 Laboratório Associado para a Sustentabilidade e Tecnologia em Regiões de Montanha (SusTEC), Instituto Politécnico de Bragança, Campus de Santa Apolónia, 5300-253 Bragança, Portugal; pjsr@ipb.pt; 3 Research Center in Digitalization and Intelligent Robotics (CeDRI), Instituto Politécnico de Bragança, 5300-253 Bragança, Portugal

**Keywords:** lemon verbena, clevenger hydrodistillation, microwave-assisted hydrodistillation, GC-MS, antioxidant activity, antimicrobial activity, anti-inflammatory activity, cytotoxicity

## Abstract

Aromatic plants are a remarkable source of natural products. *Aloysia citrodora* Paláu (Verbenaceae), commonly known as lemon verbena, is a relevant source of essential oils with potential applications due to its lemony scent and bioactive properties. Studies carried out on this species have focused on the volatile composition of the essential oil obtained by Clevenger hydrodistillation (CHD), with little information available on alternative extraction methodologies or the biological properties of the oil. Therefore, this work aimed to compare the volatile composition, antioxidant activity, cytotoxicity, anti-inflammatory and antibacterial activities of the essential oil extracted by conventional hydrodistillation by Clevenger (CHD) and Microwave-Assisted Hydrodistillation (MAHD). Significant differences (*p* < 0.05) were observed for some compounds, including the two major ones, geranial (18.7–21.1%) and neral (15.3–16.2%). Better antioxidant activity was exhibited by the MAHD essential oil in DPPH radical scavenging and reducing power assays, while no differences were observed in the cellular antioxidant assay. The MADH essential oil also presented higher inhibition against four tumoral cell lines and exhibited lower cytotoxicity in non-tumoral cells as compared with Clevenger-extracted essential oil. In contrast, the latter showed higher anti-inflammatory activity. Both essential oils were able to inhibit the growth of eleven out of the fifteen bacterial strains tested.

## 1. Introduction

Since ancient times, aromatic plants have been used both as foods and in folk medicine due to their numerous properties. Aromatic herbs produce essential oils in one or more of their botanical structures, thus frequently presenting a characteristic aroma or perfume [1]. In general, these essential oils are considered of great economic value and interest for different industries, including pharmaceuticals, cosmetics and food production. They are mainly constituted by natural and biodegradable compounds with terpene or terpenoid structures, play flavoring roles in various foods and are associated with antimicrobial properties while generally exhibiting reduced toxicity when used in very low concentrations [2]. The main essential oil-producing plant families are Apiaceae, Asteraceae, Geraniaceae, Lamiaceae, Pineaceae, Rutaceae and Verbenaceae [3]. *Aloysia citrodora* Paláu (Verbenaceae), commonly known as lemon verbena or sweet lime, is also scientifically designated by the accepted synonyms *Aloysia tryphylla* Royle, *Lippia triphylla* Kuntze, *Verbena tryphilla* L’Hér., *Lippia citrodora* (Paláu) Kunth and *Verbena citrodora* (Paláu) Cav. [4]. Lemon verbena is considered to be endemic to South America, despite being currently cultivated around the globe, particularly in North Africa and southern Europe, after being introduced in these regions by the Spanish and Portuguese in the 17th century [5]. Traditionally, the plant has been used for medicinal and aromatic purposes, namely as a folk remedy for stomach disorders, the management of insomnia and anxiety, bronchitis and heart problems, as well as for its antispasmodic and diuretic properties [5,6,7]. The biological properties of the plant have been mainly assigned to its essential oil, which generally contains geranial and neral (citral isomers) as its main compounds [6]. Due to its pleasant lemony fragrance, the plant and essential oil thereof have frequent applications in the cosmetic and food industries, particularly in the perfume industry due to their considerably high content of citral [5,6]. In addition, in some regions, the fresh or dried leaves of lemon verbena are frequently used in refreshing drinks and as a flavor in liquors, puddings and jams. Because lemon verbena essential oil is of interest to the mentioned industries, the demand for this aromatic plant has been increasing [5].

Essential oils (EOs) are frequently obtained through traditional hydrodistillation processes, such as steam distillation and hydrodistillation, which generally consume a large amount of energy, or less frequently by organic solvent extraction, which is associated with environmental drawbacks [8]. Unconventional methods such as ultrasound, microwave and enzymatic hydrolysis have recently been proposed as greener alternatives [9]. These methods mainly act by destroying the cell wall structure, resulting in a reduction in the resistance between the cell wall and the cytoplasm to solvent (water) extraction and accelerating the rate of dissolution of the active substances. Microwave-Assisted Hydrodistillation has become very popular in recent years due to the feasibility of using closed containers that allow better control of the process, reducing energy costs and time when compared with conventional extractions [10]. According to the literature, besides relevant factors that are known to impact the chemical composition of plants’ essential oils, such as edaphoclimatic conditions, genetics and collecting period, the method of extraction can also influence the composition [11,12].

In this sense, the present work aimed to compare two different techniques for extracting the essential oil of *A. citrodora* dry leaves, namely the traditional hydrodistillation by Clevenger (CHD) and Microwave-Assisted Hydrodistillation (MAHD). The obtained essential oils were evaluated according to their volatile profiles and bioactivities (antioxidant, anti-proliferative and antimicrobial), and their differences were assessed to further promote their use in the food and cosmetics industries.

## 2. Results and Discussion

### 2.1. Essential Oil Hydrodistillation and Volatile Compound Composition

Table 1 shows the results of the identification of the components of lemon verbena oil by GC-MS for the extractions by CHD and MAHD, with the respective relative percentages, while the respective chromatograms are shown in Figure S1 (Supplementary Material). The relative amounts of the individual compounds were determined based on the integration of the peaks’ areas obtained from total ion current (TIC) chromatograms. GC-MS analysis allowed the identification of 87.7% and 89.0% of the compounds in the CHD and MAHD EOs, respectively, in both cases corresponding to a total of 69 compounds identified. Therefore, the type of hydrodistillation process did not affect the qualitative profile of lemon verbena EOs, which presented citral isomers geranial (18.7–21.1%) and neral (15.3–16.2%) as the major compounds, followed by spathulenol (7.2–8.7%), caryophyllene oxide (5.3–5.6%), limonene (5.0–5.4%) and *ar*-curcumene (4.7–5.3%). Nevertheless, for some compounds, significant differences (*p* < 0.05) were found regarding their relative content according to the extraction method used. This was verified for some compounds presenting relative amounts >5%, such as geranial, neral, spathulenol and ar-curcumene, but also for others presenting much lower content, such as 1-*epi*-cubenol, *β*-curcumene, cis-sabinene hydrate, terpinene-4-ol, *γ*-terpinene or *α*-terpinene (Table 1).

Significant differences between the essential oils extracted by CHD or MADH were also verified for total oxygenated monoterpenes (49.7–53%, *p* = 0.008) and total sesquiterpenes (21.5–22.1%, *p* = 0.01). In general, the volatile chemical profile was in good agreement with previous studies that also reported citral isomers (geranial and neral) as the major compounds. Santos-Gomes et al. (2005) [14] evaluated the EOs extracted by hydrodistillation from *A. citrodora* leaves grown in Portugal and reported a profile similar to the one herein reported for a sample grown in the same country. Namely, the authors identified a total of 63 compounds, with the main volatiles being geranial (from 26.80% to 38.30%), neral (from 20.80% to 29.60%) and limonene (from 5.70% to 20.60%), which supports the citric aroma frequently associated with this species. These monoterpenes were also reported as major compounds detected in the essential oil of lemon verbena leaves grown in Greece [15], with contents ranging from 26.8 to 38.7% for geranial, from 21.8 to 24.5% for neral and from 5.8 to 17.7% for limonene. In this study, it was shown that the amount of these compounds may vary depending on the collection period, with the amount of citral isomers tending to decrease from May to September while there is an increase in limonene. A similar qualitative profile yet showing quantitative differences, with limonene being the major compound, was reported by Hudaib et al. [16] for the oil hydrodistilled from the whole aerial parts of *A. citrodora* grown in Jordan. The differences in compound quantities may be explained by the use of the whole aerial parts instead of just the leaves. Likewise, Özek et al. [17] identified 69 compounds in the oil extracted from *A. triphylla* (syn. *A. citrodora*) leaves and described a higher content of limonene (18.59%) compared to geranial (11.93%) and neral (5.99%).

Despite the fact that most works report compositions similar to those shown in Table 1, some studies have described the existence of chemotypes showing a very different volatile profile. Oukerrou et al. (2017) [18] identified 72 volatiles in the essential oil obtained from plants grown in Morocco and described high contents of sesquiterpenes, with *β*-spathulenol (9.42–15.61%), *ar*-curcumene (11.28–15.15%) and *trans*-caryophyllene oxide (13.25–14.14%) presenting higher amounts than neral (6.27–10.02%) and geranial not being detected. Gil et al. [19] concluded that the genotype is the most relevant factor affecting the essential oil composition and described three different chemotypes in different accessions grown in Argentina: the most frequent with typical lemony aroma due to citral isomers and limonene and two others, namely, one showing a preponderance of *α*-pinene, 1,8-cineol and *ar*-curcumene and low contents of citral isomers and another showing unusually high contents of sabinene, *cis*-thujone and citronellal, which are not considered to be representative of typical lemon verbena essential oil. Distinct chemotypes in samples grown in Argentina were also described by Elechosa et al. [20] and Lira et al. [21], who defined this region as being a center of *A. citrodora* genetic diversity, resulting in remarkable chemical diversity with at least four clearly defined chemotypes in northwestern Argentina. Among these chemotypes, the ones presenting a lower content of citral isomers and limonene are generally less interesting for the industry due to their detrimental aromatic profile and are usually not selected for cultivation. The sample evaluated in the present study was supplied by a producer of aromatic plants, thus presenting high contents of the desired main compounds, as expected for a plant grown for commercial purposes. When comparing the two extraction methods, MADH presented the advantages of delivering an essential oil richer in geranial and neral in a shorter time, thus spending less energy. Moreover, MADH also resulted in a higher extraction yield (1.29 ± 0.17%) compared to CHD (1.05 ± 0.07%). 

### 2.2. Bioactivity Properties

#### 2.2.1. Antioxidant Activity

In order to evaluate the antioxidant activity of the essential oils, three different in vitro assays were employed: DPPH, reducing power (RP) and cellular antioxidant activity (CAA). The results obtained are shown in Table 2.

The results of the CAA assay, which, to the best of our knowledge, has been applied for the first time to *A. citrodora* EOs, showed comparable high antioxidant activity for both EOs (*p* > 0.05). Nevertheless, significant differences (*p* < 0.05) were obtained between the two hydrodistillation methods regarding the DPPH and RP results, with lower EC_50_ values (corresponding to stronger antioxidant activity) being observed in the MADH essential oil compared to the CHD. Given the similarity of the two essential oils, the discrepancy observed in the DPPH and RP assays can be attributed to the compounds that showed quantitative differences. Thus, a linear regression analysis was performed in an attempt to identify the compounds that contribute the most to the superior antioxidant activity evidenced by the MADH essential oil in the DPPH and RP assays. Linear regression imposes some assumptions for a specific regressive modeling problem between independent and dependent variables; namely, classical linear regression assumes linear relationships between independent (explanatory) variables and the dependent (response) variable. By calculating the Pearson correlation, we found that most independent variables (volatile compounds) exhibit linearity with the corresponding dependent variables (results of the DPPH and RP assays). The remaining assumptions were also verified since the two samples (EOs extracted by two different methods) independently passed the Breusch–Pagan and Shapiro–Wilk tests. After that, elastic-net coefficients were calculated as described in the Section 3, and the obtained values are shown in Table 3. Since lower EC_50_ values correspond to higher antioxidant activity, the lower the elastic-net coefficients, the higher their contribution to explaining the better performance of the MADH sample in the antioxidant activity assays.

The applied statistical analysis highlighted sixteen compounds as the ones contributing the most to explaining the better activity of the MADH essential oil, as evidenced by their negative elastic-net coefficients, with minor compounds such as *β*-curcumene, *cis*-sabinene hydrate and *β*-bisabolene exhibiting the lowest values. The quantitative differences between the two essential oils in other minor compounds such as *α*-terpineol, *β*-caryophyllene and bicyclogermacrene, as well as major compounds such as *ar*-curcumene, geranial and neral, were also found to be relevant in explaining the lower EC_50_ of the MADH EO compared to CHD. Interestingly, the antioxidant capacity of several of these compounds has been previously reported, with special emphasis on the major compounds neral and geranial [22,23]. According to Baschieri et al. [24], the antioxidant behavior of citral isomers (neral and geranial) occurs by co-oxidation with the substrate, supported by kinetic data and evidenced by the very fast self-termination and cross-termination of the oxidative chain. Other compounds for which antioxidant properties have been described are sabinene-hydrate, α-terpineol and caryophyllene [15,25,26], which, despite being minor compounds, were also present in significantly higher amounts in the MADH EO. Moreover, other compounds with reported antioxidant activity, such as limonene and linalool [24], were present in both oils in similar amounts, thus potentially contributing to their overall activity. Finally, it should be mentioned that some compounds presenting higher contents in the CHD extracted sample, namely *α*-terpinene and *γ*-terpinene, have also been ascribed antioxidant properties [23], albeit in minor amounts in both samples.

In general, the results obtained for DPPH were consistent with previous works. Hashemi et al. [27] investigated the extraction of essential oil from *A. citrodora* using ultrasound-assisted extraction (UAE, continuous and pulsed) and hydrodistillation using a Clevenger apparatus. They reported EC_50_ values ranging from 5 to 10 mg/mL and 10 to 15 mg/mL for hydrodistilled and UAE-extracted oils, respectively. On the other hand, the RP results obtained (1.43 and 1.76 mg/mL for MADH and hydrodistillation, respectively) were lower than the EC_50_ value of 4.38 mg/mL reported by Hosseini et al. [28] for hydrodistilled *A. citrodora* essential oil from Iran. It should be noted that these values can be influenced not only by the extraction technique but also by various intrinsic and extrinsic factors such as the geographical region, harvest time and other variables.

#### 2.2.2. Cytotoxicity and Anti-Inflammatory Activity

The cytotoxic activity of the essential oils was evaluated against six different cell lines, four tumoral and two non-tumoral. The results presented in Table 4 show that both essential oils exhibited high activity against tumoral cells, which is in agreement with a previous study by Oukerrou et al. [18]. Their study reported that the essential oil of *A. citriodora* showed a very high cytotoxic effect against the P815 mouse mastocytoma tumor cell line and high-to-moderate activity against MCF7 cells.

Comparing the oils from the two hydrodistillation methods, in general, the one obtained through MADH exhibited lower GI_50_ values for tumor cell lines and higher GI_50_ values for non-tumor cell lines, although no significant differences (*p* > 0.05) were observed for the NCI-H460 and VERO cell lines. Previous studies reported antiproliferative and antitumor properties for different individual terpenes, which may be related to the activity observed in this work [29]. Citral isomers (neral and geranial) were shown to induce apoptosis in chronic lymphoid leukemia by activation of caspase-3 [30,31]. In the study of Dahham et al. [25], the sesquiterpene *β*-caryophyllene exhibited potent activity against colon cancer cells by inducing apoptosis via nuclear condensation and fragmentation pathways, including disruption of mitochondrial membrane potential. Limonene was also found in relatively high amounts (5.0–5.4%), although in this case, it did not present significant differences between the oils extracted by different methods. Limonene has been the target of several studies that have evidenced its pleiotropic activity in cancer cells, targeting several cell-signaling pathways critically related to tumor initiation, growth and angiogenesis and being able to induce cell apoptosis [32]. The capacity to induce apoptosis and/or display antiproliferative effects or inhibit signaling pathways has also been demonstrated for other monoterpenes and sesquiterpenes, such as myrcene [33], geraniol [34], thymol and carvacrol isomers [35], terpineol [15] and linalool [36]. Despite showing activity against tumoral cells, both *A. citrodora* essential oils also showed inhibitory effects against the non-tumoral VERO and PLP2 cell lines. This was particularly relevant for CHD-extracted oil on PLP2 cells, for which a very high cytotoxic effect was observed (GI_50_= 18 μg/mL). Similar results were previously reported by Oukerrou et al. [18], who also observed a cytotoxic effect of *A. citrodora* leaves essential oil on VERO cells (lowest GI_50_ of 32.90 μg/mL). 

Regarding anti-inflammatory activity, both essential oils showed promising results, as evidenced by the inhibition of nitric oxide production in RAW 264.7 macrophages (Table 4). Contrary to antioxidant and cytotoxic properties, in this assay, the best results were obtained for the Clevenger-extracted essential oil, which presented a significantly lower IC_50_ (29 μg/mL) compared to MADH (IC_50_ = 40 μg/mL). The results obtained corroborate the anti-inflammatory effect previously observed in vivo using the carrageenan-induced rat hind paw edema model for both citral and the hexane extract of *A. citrodora* [37]. The efficacy of citral isomers in inhibiting cytokine expression in murine macrophages stimulated by LPS was investigated by Liao et al. [38], who concluded that the two isomers induced different intracellular molecular responses, with neral showing more potent anti-inflammatory activity. Although these two isomers may contribute to the overall anti-inflammatory activity, they were present in lower amounts in the CHD-extracted oil, which does not correlate with its higher activity. This was also shown by applying the described linear regression analysis, which highlighted 19 compounds contributing to explaining the better activity of the Clevenger sample, with the highest contribution being attributed to spathulenol, followed by terpinene-4-ol, 1-*epi*-cubenol and *γ*-terpinene (Table 3). Previous works have indeed demonstrated the anti-inflammatory activity of spathulenol [39,40], terpinene-4-ol [41,42] and *γ*-terpinene [43]. In an assay similar to the one used in this work, Costa et al. [39] demonstrated that spathulenol was able to modulate nitric oxide production on LPS-stimulated macrophages (IC_50_ 45.6 ± 1.4 μg/mL). Hart et al. [42] demonstrated that terpinen-4-ol suppresses the production of pro-inflammatory mediators by activated human monocytes, while there is also in vivo evidence that it inhibits the production of pro-inflammatory cytokines in rats with arthritis [41]. Regarding *γ*-terpinene, its in vivo activity was reported in different models of inflammation [43]. Additionally, anti-inflammatory activity has been described for other terpenes identified in *A. citrodora* essential oil, such as limonene [44] and linalool [45], which are expected to contribute to its overall activity.

#### 2.2.3. Antibacterial Activity

The results of the antibacterial activity tested against different foodborne and clinically isolated bacteria are shown in Table 5. All the tested bacteria causing food infections or food poisoning were sensitive to the essential oil of *A. citrodora* (MICs ranging from 0.019 to 0.63%, *v*/*v*), with the exception of *Pseudomonas aeruginosa.* For this group of bacteria, the gram-negative *Yersinia enterocolitica* and gram-positive *Bacillus cereus* were the most sensitive, with a MIC of 0.08% (*v*/*v*). However, for *Y. enterocolitica*, as well as for *S. aureus*, *S. enterica* and *L. monocytogenes*, no bactericidal effect was evidenced at the highest tested concentration. Overall, the antimicrobial activity exhibited against the clinical bacteria was the worst since no activity was observed against *P. aeruginosa*, *P. mirabilis* or *K. pneumonia*, and a bactericidal effect was found only for *E. coli*. Among this group of bacteria, better inhibition of gram-positive strains was observed in relation to gram-negative strains. Interestingly, MRSA presented low MIC values that, in the case of the Clevenger essential oil, were even lower than those verified for non-methicillin-resistant *S. aureus* (0.15% versus 0.3%, *v*/*v*). The MIC values of *L. monocytogenes* and *E. coli* isolated from patients were much higher when compared to the tested ATCC strains.

In general, the essential oil extracted by Clevenger performed better when compared to that obtained by MAHD since it presented lower MIC values for 4 out of the 15 tested bacteria. The exception was *E. coli* isolated from expectoration, which showed a lower MIC for the MADH essential oil (1.25%, *v/v*) compared to the CHD EO (2.5%, *v/v*). 

The results of the present study corroborate those reported by Oukerrou et al. [18]. The authors conducted a study to characterize five essential oils extracted by hydrodistillation from *A. citrodora* plants collected from various regions in Morocco and verified their bacteriostatic and bactericidal activities against *E. coli* and *S. aureus*. In the present work, a bactericidal effect was not observed for *S. aureus*, possibly because lower concentrations were tested. Similarly, the antimicrobial activity and bactericidal effect of *A. citrodora* essential oil were demonstrated against *E. coli* and *E. faecalis*, although using a different technique (disc diffusion assay) [46,47].

## 3. Materials and Methods

### 3.1. Sample Preparation

Lemon verbena dry leaves were provided by a specialized company in Portugal (Cantinho das Aromáticas, Porto, Portugal). Before the analysis, the samples were ground to powder (model A327R1, Moulinex, Barcelona, Spain) and stored at room temperature (∼25 °C), protected from light.

### 3.2. Clevenger Hydrodistillation

The EO was extracted using a Clevenger system (Vilabo, Marinha Grande, Portugal) coupled to a 5000 mL distillation round flask, where 100 g of sample was added together with 2000 mL of distilled water. The flask was placed on a heating mantle (Nahita Blue Heating Matle, series 655, Navarra, Spain) for 180 min, counting after the first drop of essential oil was obtained. The oil was collected into a vial, where anhydrous sodium sulfate was added to eliminate any trace of water that had passed with the oil during the collection process.

### 3.3. Microwave-Assisted Hydrodistillation (MAHD)

A microwave synthesis system (NuWay-uno, Nutech Analytical Technologies Pyt. Ltd., Kolkata, India) coupled to a Clevenger apparatus was used to extract essential oil. The parameters set for the hydrodistillation were the following: 600 W potency, 15 min, 25 g of dried sample and 250 mL of distilled water in a 500 mL distillation round flask.

### 3.4. Evaluation of the Volatile Composition of the Essential Oil

A GC-2010 Plus gas chromatography system (Shimadzu, Kyoto, Japan) coupled with an AOC—20iPlus automatic injector (Shimadzu, Kyoto, Japan), a quadrupole mass spectrometry detector and a SH-RXi-5ms column (30 m × 0.25 mm × 0.25 μm; Shimadzu, Colombia, SC, USA) was used to analyze the volatile compounds. Temperature conditions, injection volume, split ratio and MS conditions were set as previously described by Spréa et al. (2020) [48]. Compounds were identified based on their mass spectra (NIST17) and calculated linear retention index (LRI) using a n-alkane compounds series (C8–C40, ref. 40147-U, Supelco) [13].

### 3.5. Bioactivity Evaluation

#### 3.5.1. Antioxidant Activity

DPPH (2,2-diphenyl-1-dicrylhydrazyl) and reducing power (RP) assays were performed as previously described [48]. The essential oils (obtained from Clevenger and Microwave-Assisted Hydrodistillation) were diluted with methanol, and 30 μL of each dilution was added to 270 μL of a methanolic solution containing a concentration of 6 × 10^−5^ mol/mL of DPPH radicals. The results were expressed as IC_50_ (mg/mL), which translates to the extract concentration providing 50% of antioxidant activity by radical scavenging. Furthermore, the essential oils were subjected to cellular antioxidant activity (CAA) and dissolved in methanol to obtain a concentration of 8 mg/mL. The CAA assay was previously described in [49].

#### 3.5.2. Cytotoxicity Activity 

The cytotoxic activity of the essential oils was evaluated according to the sulforhodamine B method [50]. Four human tumoral cell lines, namely human gastric epithelial cell line (AGS), human colorectal adenocarcinoma (CaCo2), breast carcinoma (MCF-7) and non-small cell lung cancer (NCI-H460), and two non-tumoral cell lines, PLP2 (primary pig liver cell culture) and VERO (African green monkey kidney) (Leibniz-Institute DSMZ—German Collection of Microorganisms and Cell Cultures GmbH), were used to evaluate the cytotoxic potential. Results were expressed in GI_50_ (concentration of extract with the ability to inhibit 50% of cell growth).

#### 3.5.3. Anti-Inflammatory Activity

The mouse macrophage cell line RAW 264.7 was used to determine the anti-inflammatory activity, as previously described in [51]. The essential oils were dissolved in methanol to an initial concentration of 8 mg/mL, followed by serial dilutions. The in vitro assessment was conducted using murine macrophage cells (RAW 264.7), and the procedure was carried out by measuring the inhibition of nitric oxide (NO) production. Dexamethasone was used as the positive control. Results were expressed as IC_50_ (μg/mL). 

#### 3.5.4. Antibacterial Activity

The essential oils were tested against several bacteria frequently associated with foodborne diseases, namely gram-negative *Enterobacter cloacae* (ATCC 49741)*, Escherichia coli* (ATCC 25922)*, Pseudomonas aeruginosa* (ATCC 9027)*, Salmonella enterica* (ATCC 13076) *and Yersinia enterocolitica* (ATCC 8610) and gram-positive *Bacillus cereus* (ATCC 11778), *Listeria monocytogenes* (ATCC 19111) and *Staphylococcus aureus* (ATCC 25923). Moreover, isolated clinical bacteria obtained from hospitalized patients were also assayed, namely gram-negative *Escherichia coli*, *Proteus mirabilis*, *Klebsiella pneumoniae* and *Pseudomonas aeruginosa* and gram-positive *Enterococcus faecalis*, *Listeria monocytogenes* and methicillin-resistant *Staphylococcus aureus* (MRSA). Minimum inhibitory concentrations (MIC) were determined using the broth microdilution method described by the Clinical and Laboratory Institute (CLSI) guidelines with minor modifications, namely the use of iodonitrotetrazolium chloride (INT) dye, which allows for a colorimetric measurement as described in [52].

### 3.6. Statistical Analyses

All values are presented as the mean ± standard deviation of assays performed in triplicate. To check for significant differences between the two hydrodistillation methods, an analysis of variance was performed, followed by Tukey’s test after confirmation of the homoscedasticity of the values, using the SPSS software version 28 (IBM, Armonk, NY, USA) with a significance of 0.05. 

Additionally, to find out the contribution of each identified volatile compound to the differences in antioxidant and anti-inflammatory activities observed between the two essential oils, a linear regression model for each dependent variable was performed since, statistically, it is a method that can obtain the contributions of independent variables on a dependent variable. To verify the required assumptions of classical linear regression, the Pearson correlation, Breusch–Pagan test and Shapiro–Wilk test were applied independently for the two samples (essential oils extracted by the two methodologies). In classical linear regression, it is also assumed that independent variables are not highly correlated and that only the necessary variables intervene in the model. These last two assumptions were circumvented by resorting to a variant of classical linear regression designated Elastic-net [53]. This modeling process adds two regularization elements to the classical cost function, one based on the L1 norm and the other on the L2 norm, applied to the values of the regressor coefficients. The L1 norm minimizes the number of coefficients, eliminating unimportant variables and reducing dimensionality. The L2 norm causes a reduction in the magnitude of the coefficient values, forcing a better distribution of partial contributions to the dependent variable value. The L2 norm also establishes a convex error space, guaranteeing a single minimum in the cost function and stabilizing the minimization process. The two norms used in a balanced way conveniently generalize the relationship between independent and dependent variables, even with a significantly reduced number of samples. The participation of each norm, L1 and L2, was determined by a grid-search process where the models were tested so that the determination coefficient (R^2^) was maximum but without exceeding the value of 0.995 to avoid the determination of coefficients in overfitting. The independent variables were normalized to obtain models with a mean of 0 and a standard deviation of 1. Each model was allowed to obtain an intercept value. The computational implementation was performed in Python using the sklearn module version 1.2.2 [54].

## 4. Conclusions

The essential oil extracted from *A. citrodora* leaves presented a predominance of oxygenated monoterpenes (49.7–53%), with the major compounds being geranial and neral isomers. Overall, the same qualitative profile was obtained by the two hydrodistillation methods evaluated. However, small but statistically significant (*p* < 0.05) differences were found regarding the abundance of 23 out of 69 compounds. Although an economic/cost study for both methodologies was not carried out, considering that Microwave-Assisted Hydrodistillation is significantly faster, it is expected that energy expenditure will be lower, and therefore this method is generally considered to be a “greener” option. The essential oil obtained by this method was richer in geranial and neral, which are generally associated with the lemony scent appreciated by consumers, therefore being more appealing to the cosmetics and food industries. Moreover, it showed better performance in two antioxidant activity assays and exhibited higher cytotoxicity against four lines of tumoral cells while presenting higher GI_50_ values for non-tumoral cells compared with the Clevenger hydrodistilled essential oil. This last sample showed high cytotoxicity against non-tumoral cells, particularly hepatocytes, as evidenced by its lower GI_50_ (PLP2 cells, 18 μg/mL) value, which was even lower than those obtained for the tumoral cell lines tested. In contrast, despite both essential oils exhibiting high anti-inflammatory activity, better results were found for the Clevenger-extracted one, which can be attributed to its higher relative content in spathulenol, terpinene-4-ol and *γ*-terpinene, for which this activity has previously been reported. In terms of antibacterial activity, in general, both essential oils showed similar activity as they were able to inhibit the growth of most tested bacteria and also showed bactericidal activity against *E. coli*, *E. cloacae* and *B. cereus*. In summary, the obtained results demonstrated the interesting biological properties of *A. citrodora* essential oil, highlighting its potential use beyond its organoleptic features. Nevertheless, particularly for the essential oil extracted by Clevenger, more studies are required regarding the evaluation of its hepatotoxicity, which may raise some concerns if the EO is used for internal administration. 

## Figures and Tables

**Table 1:** Identification of the constituents of the essential oil of *A. citrodora* extracted by Clevenger hydrodistillation (CHD) and Microwave-Assisted Hydrodistillation (MAHD). Results are expressed in relative percentages (%).

Peak	Compound	LRI ^a^	LRI ^b^	CHD	MAHD	
				Mean ± SD	Mean ± SD	*p*
1	(*E*)-2-Hexenal	846	846	0.0286 ± 0.0005	0.047 ± 0.004	0.019
2	*α*-Thujene	922	924	0.029 ± 0.004	0.03 ± 0.001	0.751
3	*α*-Pinene	928	932	0.21 ± 0.01	0.22 ± 0.01	0.615
4	Sabinene	968	969	0.44 ± 0.04	0.476 ± 0.007	0.309
5	*β*-Pinene	971	974	0.032 ± 0.003	0.035 ± 0.001	0.237
6	1-Octen-3-ol	975	974	1.1 ± 0.1	0.74 ± 0.03	0.044
7	6-methyl-5-Hepten-2-one	982	981	0.83 ± 0.04	0.593 ± 0.0002	0.017
8	*β*-Myrcene	987	988	0.07 ± 0.01	0.077 ± 0.002	0.374
9	3-Octanol	991	988	0.27 ± 0.03	0.206 ± 0.003	0.072
10	(2*E*, 4*E*)-Heptadienal	1005	1005	0.02 ± 0.002	0.023 ± 0.007	0.623
11	*α*-Terpinene	1011	1014	0.056 ± 0.005	0.034 ± 0.001	0.025
12	*p*-Cymene	1016	1020	0.115 ± 0.006	0.111 ± 0.006	0.575
13	Limonene	1025	1024	5.38 ± 0.06	5 ± 0.1	0.069
14	1,8-Cineole	1026	1026	4.2 ± 0.4	4.5 ± 0.2	0.391
15	(*E*)-*β*-Ocimene	1044	1044	0.0198 ± 0.0003	0.028 ± 0.006	0.185
16	*γ*-Terpinene	1054	1054	0.098 ± 0.004	0.057 ± 0.003	0.008
17	*cis*-Sabinene hydrate	1062	1065	0.412 ± 0.009	0.674 ± 0.008	0.001
18	*cis*-Linalool oxide	1067	1067	0.08 ± 0.005	0.053 ± 0.002	0.015
19	*trans*-Linalool oxide	1082	1084	0.088 ± 0.001	0.06 ± 0.02	0.196
20	Linalool	1095	1095	0.606 ± 0.008	0.64 ± 0.02	0.122
21	*trans*-*ρ*-Mentha-2,8-dien-1-ol	1115	1119	0.239 ± 0.005	0.245 ± 0.004	0.295
22	*α*-Campholenal	1121	1122	0.088 ± 0.002	0.091 ± 0.004	0.464
23	*cis*-*p*-Mentha-2,8-dien-1-ol	1130	1133	0.34 ± 0.04	0.38 ± 0.06	0.470
24	*trans*-Limonene oxide	1134	1137	0.41 ± 0.02	0.35 ± 0.03	0.097
25	*exo*-Isocitral	1140	1140	0.258 ± 0.003	0.26 ± 0.01	0.903
26	Citronellal	1148	1148	0.056 ± 0	0.055 ± 0.007	0.778
27	Nerol oxide	1150	1154	0.0481 ± 0.0004	0.032 ± 0.007	0.078
28	Sabina ketone	1152	1154	0.09 ± 0.006	0.089 ± 0.007	0.925
29	(*Z*)-Isocitral	1160	1160	0.84 ± 0.02	0.85 ± 0.04	0.655
30	*δ*-Terpineol	1162	1162	0.212 ± 0.004	0.226 ± 0.001	0.046
31	Rosefuran epoxide	1169	1173	0.61 ± 0.01	0.62 ± 0.01	0.832
32	Terpinen-4-ol	1171	1174	0.46 ± 0.01	0.252 ± 0.008	0.002
33	(*E*)-Socitral	1177	1177	1.11 ± 0.03	1.06 ± 0.04	0.323
34	*α*-Terpineol	1186	1186	1.744 ± 0.003	1.821 ± 0.005	0.003
35	Methyl chavicol	1195	1195	0.37 ± 0.01	0.351 ± 0.01	0.167
36	*trans*-Carveol	1215	1215	0.3 ± 0.02	0.3 ± 0.04	0.987
37	Nerol	1225	1127	0.96 ± 0.04	0.94 ± 0.04	0.624
38	Neral	1240	1235	15.3 ± 0.1	16.2 ± 0.1	0.011
39	Geraniol	1252	1249	0.98 ± 0.02	0.93 ± 0.02	0.110
40	Geranial	1271	1264	18.726 ± 0.007	21.1 ± 0.3	0.009
41	(*E*)-Anethole	1283	1282	1.06 ± 0.03	1.07 ± 0.03	0.773
42	Thymol	1288	1289	0.057 ± 0.005	0.067 ± 0.002	0.133
43	Carvacrol	1298	1298	0.052 ± 0.002	0.061 ± 0.005	0.170
44	Piperitenone	1337	1340	0.021 ± 0.002	0.016 ± 0.002	0.164
45	Eugenol	1352	1369	0.157 ± 0.001	0.14 ± 0.01	0.229
46	Cyclosativene	1364	1369	0.033 ± 0.006	0.033 ± 0.003	0.952
47	*α*-Ylangene	1374	1373	0.409 ± 0.006	0.46 ± 0.02	0.053
48	Geranyl acetate	1378	1379	1.527 ± 0.005	1.62 ± 0.02	0.030
49	*β*-Bourbonene	1383	1387	0.595 ± 0.006	0.52 ± 0.07	0.293
50	Methyl eugenol	1399	1403	0.14 ± 0.02	0.15 ± 0.04	0.835
51	*α*-Cedrene	1410	1410	0.379 ± 0.009	0.42 ± 0.008	0.038
52	*β*-Caryophyllene	1417	1417	0.609 ± 0.011	0.72 ± 0.01	0.011
53	*β*-Copaene	1427	1430	0.078 ± 0.003	0.08 ± 0.007	0.705
54	*allo*-Aromadendrene	1459	1458	0.507 ± 0.007	0.55 ± 0.001	0.012
55	*γ*-Muurolene	1474	1478	0.13 ± 0.01	0.135 ± 0.001	0.635
56	*ar*-Curcumene	1480	1479	4.68 ± 0.02	5.3 ± 0.04	0.003
57	*epi*-Cubebol	1492	1493	0.291 ± 0.005	0.287 ± 0.009	0.624
58	Bicyclogermacrene	1495	1500	0.167 ± 0.01	0.3 ± 0.01	0.007
59	*β*-Bisabolene	1505	1505	0.0781 ± 0.0002	0.097 ± 0.001	0.001
60	*β*-Curcumene	1508	1514	0.0672 ± 0.0009	0.128 ± 0.001	0.000
61	Cubebol	1513	1514	0.801 ± 0.001	0.94 ± 0.02	0.010
62	*α*-Cadinene	1535	1537	0.065 ± 0.005	0.1 ± 0.02	0.179
63	(*E*)-Nerolidol	1560	1561	1.76 ± 0.03	1.61 ± 0.03	0.037
64	Spathulenol	1580	1577	8.71 ± 0.01	7.23 ± 0.03	0.000
65	Caryophyllene oxide	1584	1582	5.6 ± 0.1	5.36 ± 0.05	0.134
66	1-*epi*-Cubenol	1627	1627	0.112 ± 0.004	0.049 ± 0.006	0.007
67	*epi*-*α*-Cadinol	1639	1638	1.65 ± 0.03	1.2 ± 0.2	0.070
68	Germacra-4(15),5,10(14)-trien-1-*β*-ol	1684	1694 *	0.286 ± 0.082	0.19 ± 0.06	0.317
69	Acorenone	1689	1692	0.2 ± 0.04	0.18 ± 0.05	0.642
	Monoterpenes			6.9 ± 0.1	6.8 ± 0.1	0.525
	Oxygenated monoterpenes			49.7 ± 0.1	53.0 ± 0.4	0.008
	Sesquiterpenes			22.18 ± 0.02	21.5 ± 0.1	0.010
	Oxygenated sesquiterpenes			5.0 ± 0.2	4.4 ± 0.1	0.071
	Others			4.0 ± 0.2	3.25 ± 0.02	0.042

Rt: retention time; ^a^: calculated LRI, determined against a series of n-alkanes (C7-C40); ^b^: theoretical LRI, according to the literature [13]; * identified based on NIST data (National Institute of Standards and Technology, https://webbook.nist.gov/chemistry/ (accessed on 25 January 2023).

**Table 2:** Antioxidant activity of *A. citrodora* essential oils obtained through Clevenger and Microwave-Assisted Hydrodistillation methods.

Sample	DPPH (EC_50_, mg/mL)	RP (EC_50_, mg/mL)	CAA (% Inhibition) ^a^
CHD	9.583 ± 0.005	1.768 ± 0.005	90%
MADH	8.631 ± 0.005 *	1.434 ± 0.005 *	89%

* Significant differences *p* < 0.05. ^a^: maximum concentration tested in CAA assay: 2000 μg/mL.

**Table 3:** Elastic-net coefficients calculated by applying linear regression models (independent variables presenting a coefficient value of 0 for the three dependent variables (DPPH, RP and anti-inflammatory results) are not shown).

Compound	DPPH Elastic-Net Coefficients	RP Elastic-Net Coefficients	RAW264.7 Elastic-Net Coefficients
(*E*)-2-Hexenal	−0.015223	−0.001762	0.314795
1-Octen-3-ol	0.008353	0	−0.281853
6-methyl-5-Hepten-2-one	0.01721	0.00564	−0.323882
3-Octanol	0.000103	0	−0.231008
*α*-Terpinene	0.015024	0.003772	−0.311129
Limonene	0	0	−0.23009
*γ*-Terpinene	0.02079	0.009779	−0.334364
*cis*-Sabinene hydrate	−0.022256	−0.010034	0.342061
*cis*-Linalool oxide	0.017437	0.005304	−0.326583
Linalool	0	0	0.108244
*trans*-Limonene oxide	0	0	−0.145299
Nerol oxide	0	0	−0.20581
*δ*-Terpineol	−0.008497	0	0.246
Terpinen-4-ol	0.022046	0.00977	−0.339853
*α*-Terpineol	−0.021369	−0.008844	0.338362
Neral	−0.018512	−0.005719	0.331411
Geraniol	0	0	−0.115558
Geranial	−0.019105	−0.006118	0.331229
Thymol	0	0	0.063287
Piperitenone	0	0	−0.042122
*α*-Ylangene	−0.008804	−0.000167	0.250177
Geranyl acetate	−0.015402	−0.005916	0.290529
*α*-Cedrene	−0.01271	−0.00256	0.263237
*β*-Caryophyllene	−0.020263	−0.00937	0.318878
*allo*-Aromadendrene	−0.018943	−0.006705	0.316855
*ar*-Curcumene	−0.021384	−0.008915	0.34047
Bicyclogermacrene	−0.019798	−0.007066	0.330141
*β*-Bisabolene	−0.021733	−0.009353	0.34362
*β*-Curcumene	−0.022332	−0.010217	0.345083
Cubebol	−0.018454	−0.005364	0.329753
(*E*)-Nerolidol	0.013095	0.002891	−0.267416
Spathulenol	0.022694	0.011	−0.345966
Caryophyllene oxide	0	0	−0.069749
1-*epi*-Cubenol	0.021318	0.010421	−0.336428
*epi*-*α*-Cadinol	0.00373	0	−0.207589

**Table 4:** Cytotoxicity and anti-inflammatory potential of *A. citrodora* essential oil obtained through Clevenger and Microwave-Assisted Hydrodistillation methods.

	MAHD (GI_50_ μg/mL)	CHD (GI_50_ μg/mL)	Ellipticine GI_50_ μg/mL)	Dexamethasone (GI_50_ μg/mL)
Cytotoxicity potential				
AGS	42 ± 4	55 ± 1 *	1.23 ± 0.03	-
CaCo2	49 ± 5	62 ± 2 *	1.21 ± 0.02	-
MCF-7	54 ± 4	88 ± 7 *	1.02 ± 0.02	-
NCI-H460	95 ± 1	84 ± 8	1.01 ± 0.01	-
PLP2	72 ± 1	18 ± 1 *	1.4 ± 0.1	-
VERO	68 ± 6	60 ± 1	1.41 ± 0.06	-
Anti-inflammatory potential (GI_50_ μg/mL)				
RAW 264.7	40 ± 1	29 ± 3 *	-	6.3 ± 0.4

* Significant difference *p* < 0.05.

**Table 5:** Antibacterial activity of *A. citrodora* essential oils against foodborne and clinically isolated bacteria.

Foodborne Bacteria	Sample (%, *v*/*v*)				Positive Control (mg/mL)					
	CHD		MAHD		Streptomycin		Methicillin		Ampicillin	
	MIC	MBC	MIC	MBC	MIC	MBC	MIC	MBC	MIC	MBC
Gram-negative Bacteria										
*Enterobacter cloacae*	0.16	1.25	0.019	0.63	0.007	0.007	n.t.	n.t.	0.15	0.15
*Escherichia coli*	0.16	2.5	0.16	2.5	0.01	0.01	n.t.	n.t.	0.15	0.15
*Pseudomonas aeruginosa*	>2.5	>2.5	>2.5	>2.5	0.06	0.06	n.t.	n.t.	0.63	0.63
*Salmonella enterica*	0.63	>2.5	0.63	>2.5	0.07	0.007	n.t.	n.t.	0.15	0.15
*Yersinia enterocolitica*	0.08	>2.5	0.16	>2.5	0.007	0.007	n.t.	n.t.	0.15	0.15
Gram-positive Bacteria										
*Bacillus cereus*	0.08	2.5	0.16	>2.5	0.007	0.007	n.t.	n.t.	n.t.	n.t.
*Listeria monocytogenes*	0.31	>2.5	0.31	>2.5	0.007	0.007	n.t.	n.t.	0.15	0.15
*Staphylococcus aureus*	0.31	>2.5	0.31	>2.5	0.007	0.007	0.007	0.007	0.15	0.15
**Clinically Isolated Bacteria**	**Sample**				**Positive Control**					
	**CHD**		**MAHD**		**Streptomycin**		**Methicillin**		**Ampicillin**	
	**MIC**	**MBC**	**MIC**	**MBC**	**MIC**	**MBC**	**MIC**	**MBC**	**MIC**	**MBC**
Gram-negative Bacteria										
*Escherichia coli*	2.5	2.5	1.25	2.5	<0.15	<0.15	<0.0078	<0.0078	n.t.	n.t.
*Klebsiella pneumoniae*	>2.5	>2.5	>2.5	>2.5	10	>10	<0.0078	<0.0078	n.t.	n.t.
*Proteus mirabilis*	>2.5	>2.5	>2.5	>2.5	<0.15	<0.15	<0.0078	<0.0078	n.t.	n.t.
*Pseudomonas aeruginosa*	>2.5	>2.5	>2.5	>2.5	>10	>10	0.5	1	>10	>10
Gram-positive Bacteria										
*Enterococcus faecalis*	1.25	>2.5	1.25	>2.5	<0.15	<0.15	n.t.	n.t.	<0.0078	<0.0078
*Listeria monocytogenes*	1.25	>2.5	1.25	>2.5	<0.15	<0.15	<0.0078	<0.0078	n.t.	n.t.
MRSA	0.15	>2.5	0.3	>2.5	<0.15	<0.15	n.t.	n.t.	0.25	0.5

EOs were tested in the concentration range of 2.5% to 0.001% (*v/v*); n.t.: not tested; MAHD: Microwave-Assisted Hydrodistillation; CHD: Clevenger hydrodistillation.

## Data Availability

Data are available in the manuscript.

## Supplementary Materials

The following supporting information can be downloaded at: https://www.mdpi.com/article/10.3390/molecules28114528/s1, Figure S1. GC-MS chromatograms of *A. citrodora* essential oils obtained by Clevenger (A) and microwave-assisted (B) hydrodistillation methods.

## Sample Availability

The samples are not available from the authors.
