# Supplementary material for: Volatile Compounds and Biological Activity of the Essential Oil of Aloysia citrodora Paláu: Comparison of Hydrodistillation and Microwave-Assisted Hydrodistillation
Source: Molecules. 2023 Jun 2;28(11):4528. doi: 10.3390/molecules28114528 (PMC10254266; doi:10.3390/molecules28114528)
Supplement: Supplementary file 1 [file molecules-28-04528-s001.zip › molecules-2404548-SI.pdf]

molecules

MDPI

*Supplementary Material*

# Volatile compounds and biological activity of the essential oil of *Aloysia citrodora* Paláu: comparison of hydrodistillation and microwave assisted hydrodistillation

**Rafael M. Sprea[1,2], Luís H. M. Fernandes[1,2], Tânia C. S. P. Pires[1,2], Ricardo C. Calhelha[1,2], Pedro J. Rodrigues[2,3] and Joana S. Amaral[1,2,*]**

1 Centro de Investigação de Montanha (CIMO), Instituto Politécnico de Bragança, Campus de Santa Apolónia, 5300-253 Bragança, Portugal
2 Laboratório Associado para a Sustentabilidade e Tecnologia em Regiões de Montanha (SusTEC), Instituto Politécnico de Bragança, Campus de Santa Apolónia, 5300-253 Bragança, Portugal
3 Research Center in Digitalization and Intelligent Robotics (CeDRI), Instituto Politécnico de Bragança, Bragança, 5300-253, PortugalAffiliation 1; e-mail@e-mail.com
* Correspondence: jamaral@ipb.pt; Tel.: +351-273-303219

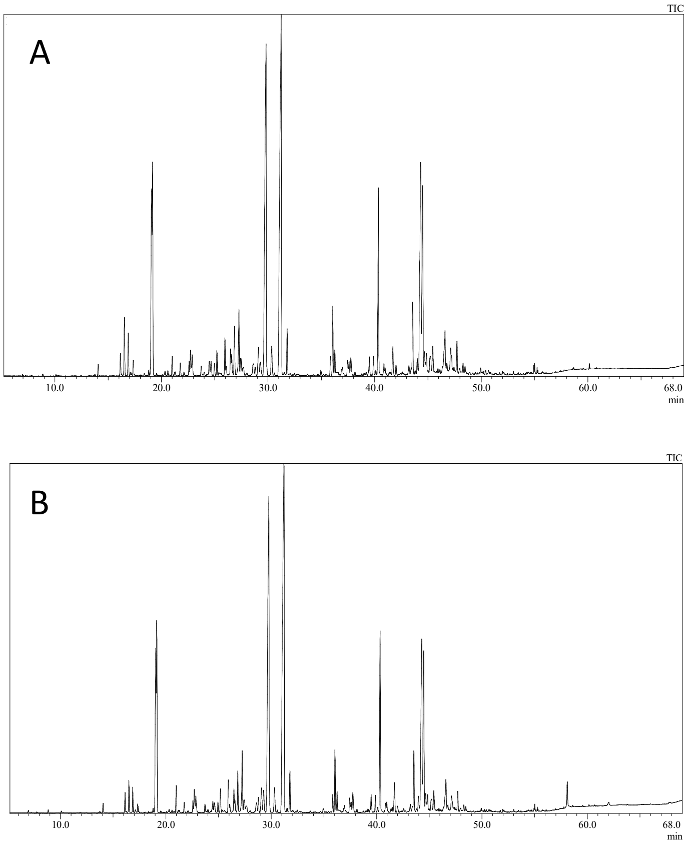


**Figure S1.** GC-MS chromatograms of *A. citrodora* essential oils obtained by Clevenger (A) and microwave (B) hydrodistillation methods.
